# Reply to: Moving the goal post and using ethics as a rhetorical refuge doesn’t change the evidence: About the debate on the best care for minors with gender issues

**DOI:** 10.1186/s12910-026-01632-y

**Published:** 2026-10-12

**Authors:** Skye Stanwich, Ezra D. Oosthoek, Karl Gerritse, David Matthew Doyle, Annelou L. C. de Vries

**Affiliations:** 1 Center of Expertise on Gender Dysphoria (CEGD), Amsterdam UMC, Vrije Universiteit, De Boelelaan 1118, Amsterdam, 1081 HZ The Netherlands; 2 Department of Child and Adolescent Psychiatry, Amsterdam UMC, Vrije Universiteit, De Boelelaan 1118, Amsterdam, 1081 HZ The Netherlands; 3 Department of Medical Psychology, Amsterdam UMC, Vrije Universiteit, De Boelelaan 1118, Amsterdam, 1081 HZ The Netherlands

**Keywords:** Gender-affirming medical treatment, Transgender adolescents, Treatment outcomes, Trans negativity

We appreciate Zepf et al. [1] taking interest in our paper [2], as well as this opportunity to clarify the aims and scope of our analysis. Our original article examined how “effectiveness” has been conceptualized and operationalized in the empirical literature on gender-affirming medical treatment (GAMT) for adolescents, specifically within the evidence base cited in the World Professional Association for Transgender Health (WPATH) Standards of Care 8 [3]. In this response, we restate the central claims of our article, clarify points as needed, and briefly reflect on ongoing discussions about evidence and outcomes in this field.

First, to clarify, we do not argue that traditional outcomes such as gender dysphoria, mental health, global functioning, or quality of life are irrelevant to adolescent transgender care. Rather, we question how these particular outcomes are established as the primary indicators of “successful” treatment, and aim to highlight the normative assumptions underlying these measures that are often taken for granted. In this sense, our original paper aims to address questions regarding how effectiveness and improvement are defined, operationalized, and evaluated in the first place, and by whom. Importantly, we do not propose replacing established clinical outcomes with concepts such as autonomy or embodiment. Our discussion of autonomy is intended to foreground the ethical relevance of trans youths’ own experiences and interpretations, both within clinical care and research. In line with this, we advocate for greater inclusion of trans youth in shaping research agendas and identifying which outcomes of GAMT are meaningful in their lived experience. Such participatory approaches are not intended to replace clinical or ethical considerations, but to complement and enrich them [4, 5].

Similarly, we would like to revisit our use of trans negativity as an analytical lens. A trans negativity perspective highlights the normative and curative idea that negative affect is a problem that should be resolved through GAMT [6, 7]. We do not propose trans negativity as a new clinical paradigm to organize adolescent care, nor do we deny the importance of alleviating suffering and supporting well-being through GAMT. Rather, trans negativity functions as a conceptual framework in our analysis for examining how dominant narratives shape expectations around transition and successful treatment outcomes of adolescent transgender care. Specifically, we identified what we termed a “logic of improvement”: a tendency within the clinical academic literature on GAMT to frame successful transition as a largely linear journey, moving from distress toward quantifiable improvement across a range of standardized psychological and psychosocial measures. Our argument is not that improvement is unimportant, but that experiences of transition may be more complex, ambivalent, or non-linear than this framework readily accommodates. Young people may continue to experience forms of distress, uncertainty, or evolving self-understanding during or after transition-related care, and still, GAMT can remain meaningful or beneficial to them. Recognizing this complexity does not undermine clinical care; instead, we believe it may support more honest communication during consultations, clearer expectation-setting, and a broader understanding of what care experiences can involve.

This latter point also bears on the discussion of beneficence and non-maleficence raised by Zepf et al. We agree that these principles are central to adolescent healthcare, including transgender care. However, these principles do not function independently of broader assumptions about risk, benefit, and uncertainty, and indeed rely on particular interpretations. For example, considerations of harm apply not only to medical interventions, but also to delayed, deferred, or withheld care. Likewise, it is important to note that non-medical interventions also operate under conditions of limited evidence [8]. In this regard, “waiting” is not simply the absence of intervention, a safe(r) first-line option, or a neutral baseline; it is a clinical and ethical choice with its own potential benefits and risks [9]. If non-medical approaches, such as psychotherapy, were associated with worsening mental health or functioning in treatment-eligible adolescents, this would equally raise concerns under the principles of beneficence and non-maleficence.

The broader aim of our article was therefore not to reject research on outcomes of GAMT, but to encourage greater reflection on how these outcomes are selected, interpreted, and prioritized within adolescent transgender care. Through the lens of trans negativity, we examined how particular understandings of successful transition become dominant within research and clinical discourse, and what experiences may become less visible as a result. We agree with Zepf et al. that gender-affirming medical care should benefit young people overall and that it should not be understood as a “cure-all” for global psychological or social difficulties. Our point is that recognizing and attending to the complexity and variability of young people’s experiences of GAMT may support more individualized and responsive care, that may include both medical and psychosocial interventions if indicated.

Research in this field continues to evolve and a growing body of work is already exploring how treatment outcomes might be conceptualized differently or more expansively, in line with a broader movement toward patient-reported outcomes in medicine [10]. This includes research using participatory methods to examine what trans people themselves consider meaningful outcomes of care [11–13], including for adolescents [14]. We view these developments as valuable contributions to ongoing conversations about evidence, effectiveness, and ethical practice in adolescent transgender care. At the same time, we emphasize that GAMT should not be completely halted pending some future ideal evidence base, particularly given the lack of clarity regarding exactly what evidence and outcome measures would be sufficient. This brings us back to one of the central points raised in our original article: what counts as adequate evidence, for whom, and under what conditions? These are not solely empirical questions; they also require ethical and conceptual reflection on how effectiveness is defined, whose perspectives are included in this process, and what forms of flourishing or difficulty are acknowledged. These are questions we believe remain important for the field in moving forward.

## Data Availability

No datasets were generated or analysed during the current study.

## Abbreviations

GAMT: Gender-affirming medical treatment
WPATH: World Professional Association for Transgender Health
